# Green Synthesis of Zinc Oxide Nanoparticles Using Puerarin: Characterization, Antimicrobial Potential, Angiogenesis, and *In Ovo* Safety Profile Assessment

**DOI:** 10.3390/pharmaceutics16111464

**Published:** 2024-11-16

**Authors:** Sergio Liga, Raluca Vodă, Lavinia Lupa, Cristina Paul, Nicoleta Sorina Nemeş, Delia Muntean, Ștefana Avram, Mihaela Gherban, Francisc Péter

**Affiliations:** 1 Department of Applied Chemistry and Engineering of Organic and Natural Compounds, Faculty of Chemical Engineering, Biotechnologies and Environmental Protection, Politehnica University Timisoara, Vasile Pârvan No. 6, 300223 Timisoara, Romania; sergio.liga96@gmail.com (S.L.); francisc.peter@upt.ro (F.P.); 2 Department of Applied Chemistry and Environmental Engineering and Inorganic Compounds, Faculty of Chemical Engineering, Biotechnologies and Environmental Protection, Politehnica University Timisoara, Vasile Pârvan No. 6, 300223 Timisoara, Romania; raluca.voda@upt.ro (R.V.); lavinia.lupa@upt.ro (L.L.); 3 Renewable Energy Research Institute-ICER, Politehnica University Timisoara, Gavril Musicescu Street No. 138, 300501 Timisoara, Romania; nicoleta.nemes@upt.ro; 4 Multidisciplinary Research Center on Antimicrobial Resistance, Department of Microbiology, Faculty of Medicine, “Victor Babeș” University of Medicine and Pharmacy, 2nd Eftimie Murgu Sq., 300041 Timisoara, Romania; muntean.delia@umft.ro; 5 Department of Pharmacognosy, Faculty of Pharmacy, “Victor Babeș” University of Medicine and Pharmacy, Eftimie Murgu Square, No. 2, 300041 Timisoara, Romania; stefana.avram@umft.ro; 6 National Institute for Research and Development in Electrochemistry and Condensed Matter, P. Andronescu Street, No. 1, 300224 Timisoara, Romania; mihaelabirdeanu@gmail.com

**Keywords:** puerarin, zinc oxide nanoparticles, metallic nanoparticles, green synthesis, CAM assay

## Abstract

Background: Zinc oxide nanobiocomposites were successfully synthesized using a green synthesis approach. The process involves the utilization of the isoflavone puerarin, resulting in the formation of PUE-ZnO NPs. Methods: Physico-chemical and biological characterization techniques including X-ray dif-fraction (XRD), UV-vis spectroscopy, Fourier transform infrared spectroscopy (ATR-FTIR), scanning electron microscopy (SEM), atomic force microscopy (AFM), and in ovo methods were employed to study the main characteristics of this novel hybrid material. Results: The PUE-ZnO NPs were confirmed to have been successfully synthesized with a UV absorption peak at 340 nm, the XRD analysis demonstrating their high purity and crystallinity. The energy band-gap value of 3.30 eV suggests possible photocatalytic properties. Both SEM and AFM images revealed the nanoparticle`s quasi-spherical shape, roughness, and size. Good tolerability and anti-irritative effects were recorded in ovo on the chorioallantoic membrane (CAM). Conclusions: According to these results, the synthesis of green PUE-ZnO NPs may be a promising future approach for biomedical and personal care applications.

## 1. Introduction

Nanotechnology and nanoscience research have been leading the way in technological advancements and have experienced significant growth over time [1,2]. Nanotechnology provides opportunities for the production of materials, particularly those for medical and pharmaceutical purposes, where conventional approaches may be at their limits [1].

The term ‘nano’ is typically used to describe particles of matter that are between 10 and 100 nm in diameter, but nanoparticles can be categorized by three size ranges: (i) between 1 and 100 nm; (ii) between 100 and 500 nm; and (iii) larger than 500 nm [3,4,5]. Their unique physical and chemical properties (e.g., surface functionalization, large surface area, tunable porosity) and their increased bioavailability allow them to be utilized as perfect candidates in biomedicine or technology to enhance efficiency and device functionality [6,7,8]. There is a range of nanoparticles available, which include magnetic nanoparticles, carbon nanoparticles, metal-based nanoparticles, lipid-based nanoparticles (e.g., liposomes, niosomes), and polymeric nanoparticles [6,7,9].

Metal-based nanomaterials are used in various fields, including biomedicine, pharmaceutical, cosmetic, biotechnology, catalysis, wastewater treatment, optical sensors, energy storage, and others [6,10,11,12,13,14]. The value of metal-based nanoparticles or metal-based nanostructures is due to their high stability, simple preparation methods, and excellent engineering control over aspects such as size, shape, porosity, surface properties, easy incorporation into hydrophobic and hydrophilic systems, and target cellular penetration capability [11,12,15].

Zinc oxide (ZnO), one of the many varieties of metal oxide nanomaterials, is one of the most widely used metal oxides due to significant features like versatility, nontoxicity, and biocompatibility, which can be customized based on its size, shape, morphology, and surface properties [15,16,17]. Zinc oxide nanoparticles are commonly found in commercial products like sunscreens, ointments, food packaging, and personal care products and have mainly gained popularity in the cosmetic industry as a result of their aesthetic and functional properties, particularly their ability to reflect visible light (e.g., physical sun-blocks or anti-UVA and anti-UVB) [18,19]. Therefore, zinc oxide nanoparticles are used in sunscreens and creams to provide effective UV protection [18]. Cosmetic technology’s preference for a clear appearance is the reason that makes the transparency characteristic of zinc oxide nanoparticles so popular and beneficial for consumers [19,20]. Other potential applications of zinc oxide nanoparticles include gene delivery, biosensors, and cancer therapy [15,17,21].

Synthesis of zinc oxide nanoparticles can be achieved using a variety of methods, with the main focus on bottom-up and top-down techniques, using various precursor ZnO salts (e.g., zinc acetate, zinc sulfate, zinc nitrate, zinc chloride) [22,23]. Furthermore, numerous studies have demonstrated that zinc oxide nanoparticles can be produced through the use of natural compounds, such as flavonoids (e.g., rutin, curcumin, quercetin, chrysin), which can add biological effects [24,25,26,27]. The scientific reason behind this is that flavonoids are well known for their affinity for metal ions, and when exposed to them can act as reducing agents, due to the abundance of hydroxyl groups, to generate ZnO nanoparticles [28,29,30]. Moreover, the flavonoids can also be incorporated with nanoparticles to improve their bioavailability and create systems with integrated functions, even though they have a low solubility, poor absorption, and rapid metabolism [28,30].

Phytoestrogens, also known as isoflavones, are plant-derived flavonoids that share structural similarities with the hormone estrogen [31,32]. The phytochemistry of isoflavones is becoming increasingly important due to their potential for having both estrogenic and antiestrogen effects on the human body [31,32,33]. Puerarin, which is also known as daidzein-8-C-glucoside, an isoflavone found in the roots of the Chinese kudzu plant, possess a variety of biological effects in acute and chronic diseases [34,35]. Efforts are underway to overcome the issues of limited bioavailability, low solubility, and low lipid stability in order to utilize puerarin as a therapeutic agent. Researchers have come up with a solution to the mentioned issues by encapsulating different nanoparticles with puerarin, mainly polymeric nanoparticles [34,36,37,38]. Metallic nanoparticles coated with puerarin were reported as efficient for various biomedical applications. For example, puerarin-coated gold nanoparticles have been successfully used for the detection of ciprofloxacin [39].

**Figure 1:**
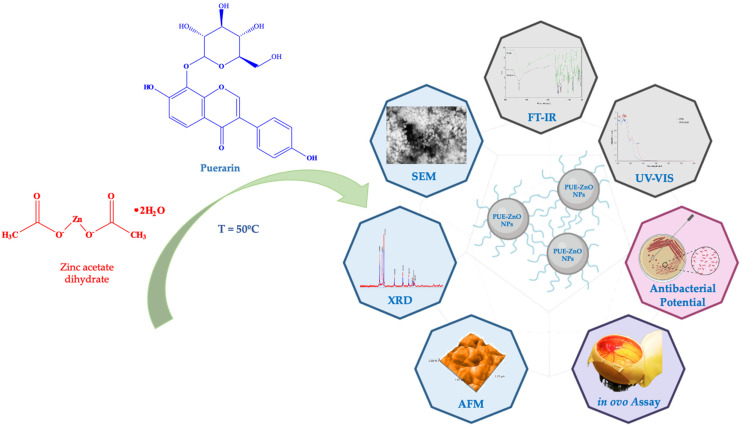
A schematic figure of puerarin-loaded ZnO nanoparticles and a summary of the techniques investigated.

In this context, the purpose of the current study was (i) green synthesis and characterization screening of puerarin-functionalized zinc oxide nanoparticles (abbreviated in our study as PUE-ZnO NPs), without the use of any additional reducing agents; (ii) antimicrobial potential evaluation; and (iii) assessment of in ovo angiogenic and anti-irritative potential (Figure 1). To our knowledge, there is no other report on the synthesis of zinc oxide nanoparticles using puerarin, physicochemical characterization, antimicrobial potential, and in ovo effects of such nanobiocomposites. Various applications can benefit from the synthesis of ZnO nanoparticles by using puerarin, including pharmaceutical and cosmetic products, cancer therapy, drug delivery, and photocatalytic materials.

## 2. Materials and Methods

### 2.1. Chemicals and Microbial Strains

Zinc acetate dihydrate (EP grade) was purchased from CHIMREACTIV SRL (Bucharest, Romania), and puerarin was obtained from BYOSINTH (CAS [3681-99-0], Bratislava, Slovakia). The analysis and quantitative and qualitative measurements involved the following chemicals and materials: dimethyl sulfoxide (DMSO) ≥ 99.5% GC (Sigma Aldrich, St. Louis, MO, USA), five microbial strains (Thermo Scientific, Waltham, MA, USA): *Staphylococcus aureus* ATCC 25923, *Streptococcus pyogenes* ATCC 19615, *Escherichia coli* ATCC 25922, *Pseudomonas aeruginosa* ATCC 27853, and *Candida parapsilosis* ATCC 22019, Columbia agar supplemented with 5% sheep blood (Oxoid, Wesel, Germany), Sabouraud agar with chloramphenicol (Oxoid, Wesel, Germany), culture mediums Mueller–Hinton agar and Mueller–Hinton fastidious agar (bioMérieux, Marcy-l’Etoile, France).

### 2.2. Green Synthesis and Characterization of Zinc Oxide Nanoparticles Using Puerarin (PUE-ZnO NPs)

**Figure 2:**
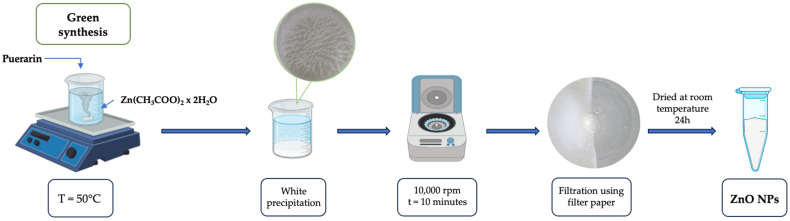
A schematic protocol of green synthesis of zinc oxide nanoparticles.

Briefly, 50 mL of preheated puerarin solution was added into a flask containing 50 mL of zinc acetate dihydrate solution. After 60 min of heating the solution with a magnetic stirrer at 50 °C, it turned milky white, which is a sign of particle formation, and then it uncolored because of particle precipitation. After this, the solution was centrifuged at 10,000 rpm for 10 min, then the supernatant was discarded. The precipitate was collected, washed with distilled water five times to remove any impurities, and dried at room temperature for 24 h (Figure 2). The resulting dried product (PUE-ZnO NPs) was stored for further analysis.

### 2.3. Characterization of Green Synthesized PUE-ZnO NPs

The characterization of the PUE-ZnO nanoparticles was performed using ultraviolet-visible (UV-vis) spectroscopy (model: UviLine 9400 Spectrophotometer, SI Analytics, Deutschland, Germany), in a scanning wavelength range of 200–800 nm, to provide a preliminary confirmation of zinc oxide nanoparticles. Fourier transform infrared spectroscopy, FT-IR (model: Bruker Vertex 70 spectrophotometer, Bruker Daltonik GmbH, Bremen, Germany, equipped with a Platinium ATR spectrometer, Bruker Diamond Type A225/Q.I), was performed, and for each sample, 128 scans were recorded in the 4000–400 cm^−1^ range.

Using X-ray diffraction (XRD), the powder patterns were achieved with a PAnalytical X’Pert MPD diffractometer, using Ni-filtered CuKα radiation (λ = 1.5418 Å), with a scan step of 0.01°, a counting time of 20 s/step, for 2θ ranged between 20° and 80°, and the powdered sample was fine-grounded by hand using an agate mortar and placed in a glass sample holder with a 20 × 20 mm square, 0.5 mm deep recess.

Scanning electron microscopy (SEM) (model: Quanta Feg 250 instrument, FEI, Eindhoven, The Netherlands), which was equipped with energy dispersive X-ray analysis (EDX), was used to identify the surface morphology, size, and elemental composition.

The topography of the PUE-ZnO NPs was investigated by Atomic Force Microscopy (AFM) using a Nanosurf^®^ EasyScan 2 Advanced Research AFM (Liestal, Switzerland). The sample was ground in a mortar with ethanol and then transferred onto a silica glass plate. It was then dried at a temperature of 25 °C. Non-contact mode was used to record the two-dimensional (2D) and three-dimensional (3D) images, with a scanned surface measuring 1.1 μm × 1.1 μm. Nanosurf EasyScan 2 computer software was used to calculate roughness parameters, which included average surface roughness (Sa, nm), maximum peak height (Sp, nm), and maximum valley depth (Sv, nm).

### 2.4. In Vitro Evaluation of the Antibacterial Activity

Green-synthesized ZnO nanoparticles were evaluated for their antimicrobial activity against five microbial strains (Thermo Scientific, USA). Due to their prevalence in healthcare-associated infections and their ongoing challenges in the biomedical field, the five pathogenic bacterial strains (Gram-negative and Gram-positive bacteria, yeast) were selected and used for testing: *Staphylococcus aureus* ATCC 25923, *Streptococcus pyogenes* ATCC 19615, *Escherichia coli* ATCC 25922, *Pseudomonas aeruginosa* ATCC 27853, and *Candida parapsilosis* ATCC 22019.

The antimicrobial activity was evaluated in accordance with the recommendations set forth by the European Committee on Antimicrobial Susceptibility Testing (EUCAST) [40] and the Clinical Laboratory Standard Institute (CLSI) [41]. Isolation of all bacterial strains was performed on Columbia agar with 5% sheep blood, while Sabouraud agar with chloramphenicol was utilized for *Candida parapsilosis*. The concentration of 0.5 McFarland was achieved by preparing the microbial suspensions with 0.85% NaCl.

#### Determination of the Minimum Inhibitory Concentrations by Dilution Method

A series of dilutions of the tested compounds were performed, with concentrations ranging from 100 to 6.25 mg/mL. In each test tube, 100 µL of each dilution of the PUE-ZnO NPs, 50 µL of Mueller–Hinton agar/Mueller–Hinton fastidious agar and 50 µL of the microbial suspension were added. The minimum inhibitory concentration (MIC) was determined as the lowest concentration exhibited by PUE-ZnO NPs without visible bacteria growth after 24 h at 35 °C [42,43].

### 2.5. In Ovo CAM Assay

The in ovo study was used to assess the potential irritability of the tested samples, as well as to establish if the samples can interfere with the angiogenesis process. For this purpose, the chorioallantoic membrane basic protocol [44] adapted to our laboratory was implemented. Briefly, the process consisted of incubating fertilized chicken eggs (*Gallus gallus domesticus*) at 37 °C in a humidified atmosphere, until the removal of albumin, followed by the opening and subsequent resealing of the upper eggs’ shells.

The irritability test was carried out using the Hen’s Egg Test–Chorioallantoic Membrane Protocol [45]. As part of the method, the controls utilized are 0.5% sodium dodecyl sulfate (positive control) and distilled water (negative control). During this experiment, test samples in a concentration of 100 μg/mL, next to the positive control and negative control, were placed on the chorioallantoic membrane on day 9 of incubation. The vascular plexus was examined under a stereomicroscope for 300 s to observe vascular change. An irritation score was calculated by analyzing the incidence of vascular events, which enabled us to classify the tested samples into one of the following classifications, according to Luepke [46]: (i) non-irritant (0 ÷ 0.9); (ii) weak irritant (1 ÷ 4.9); (iii) moderately irritant (5 ÷ 8.9); and (iv) strongly irritant (8.9 ÷ 21). The irritability score was calculated using the following formula (Equation (1)) [47]:

(1) $$IS=5\times \frac{301-{Sec}_{H}}{300}+7\times \frac{301-{Sec}_{L}}{300}+9\times \frac{301-{Sec}_{C}}{300}$$

where the vascular event is measured in seconds and outlined as follows: ${Sec}_{H}$—hemorrhage event, ${Sec}_{L}$—lysis event, and ${Sec}_{C}$—coagulation event.

To assess the changes upon the angiogenesis process, puerarin and PUE-ZnO NPs were prepared with 0.5% DMSO to achieve concentrations of 100 μg/mL, and then 10 μL was placed inside plastic rings located on top of vascularized areas of the chorioallantoic membrane on day 7 of incubation.

The potential vascular changes that occurred due to the tested samples were monitored using stereomicroscopic live analysis and imaging (ZEISS SteREO Discovery.V8, Göttingen, Germany), coupled to a camera (Axiocam 105 color, AxioVision SE64. Rel. 4.9.1 Software, (ZEISS, Göttingen, Germany), while the selected images were processed by ImageJ (https://imagej.net, accessed on 6 July 2024).

## 3. Results and Discussion

ZnO NPs were successfully synthesized for the first time by using the isoflavone puerarin and a synthesis route that meets the principles of Green Chemistry. At 50 °C, a zinc acetate solution was added to puerarin and stirred continuously until it turned milky white and then uncolored, indicating the formation of ZnO NPs. The 1:1 optimal molar ratio between the concentrations of the precursors was achieved through multiple experiments. ZnO NPs’ green production is firstly indicated by a change in the reaction mixture’s color, followed by precipitation. A mechanism proposed in the literature for the synthesis of flavonoid–metal nanoparticles states the formation of coordination bonds between isoflavones and nanoparticles, since flavonoids can act as hydrogen donors and create stable metal coordination complexes [48,49,50]. As it will result from the following physico-chemical investigations, our study demonstrated that the isoflavone puerarin mediated the reduction process and the stabilization of the nanoparticles, also attaching itself to the ZnO NPs. Compared to other reports, a greener pathway was accomplished by using only the isoflavone as a reducing agent in the nanoparticle synthesis process, without a hydoxyde or other chemicals, as well as by drying the nanoparticles at room temperature and consequently reducing the energy consumption. A comprehensive characterization of the resulted nanoparticles was achieved to reveal their structural and morphological properties and demonstrate the inclusion of puerarin in the resulted nanobiocomposite material, targeting a possible synergic effect of both these components in various applications.

### 3.1. Physicochemical Characterization of ZnO NPs Synthesized by a Green Chemistry Pathway

#### 3.1.1. X-Ray Diffraction (XRD) Analysis

**Figure 3:**
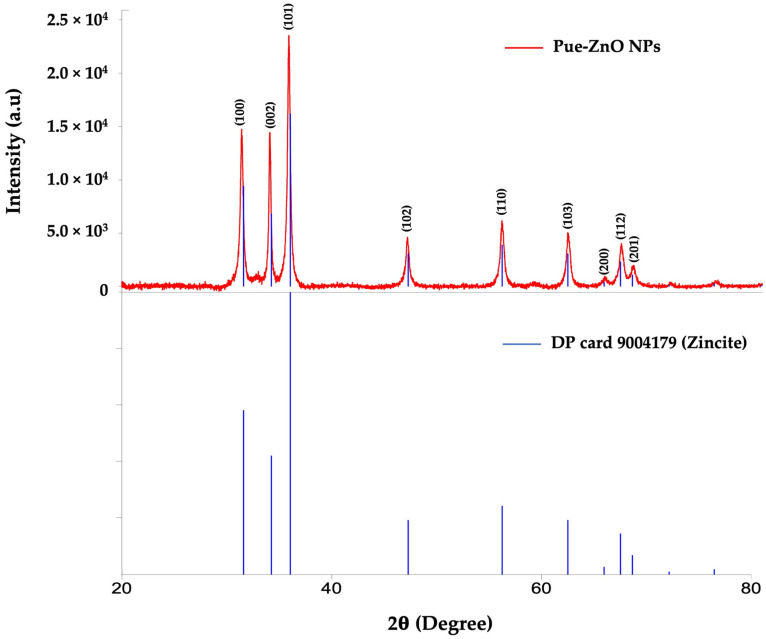
XRD patterns of the ZnO NPs synthesized by a green pathway using puerarin at 50 °C.

The phase identification and crystalline structure of the ZnO NPs synthesized by using puerarin were confirmed by X-ray diffraction. All diffraction peaks were matched with DP card 9004179. Figure 3 shows the XRD pattern of the PUE-ZnO NPs, with several sharp and strong diffraction peaks predicted at different 2θ values 31.39°, 34.07°, 35.86°, 47.19°, 56.21°, 62.50°, and 66.06°, corresponding to different Miller indices (110), (002), (101), (102), (110), (103), (112), indicating a typical zincite ZnO structure diffraction. Peaks corresponding to impurities were not observed.

#### 3.1.2. UV-Visible Spectroscopy

**Figure 4:**
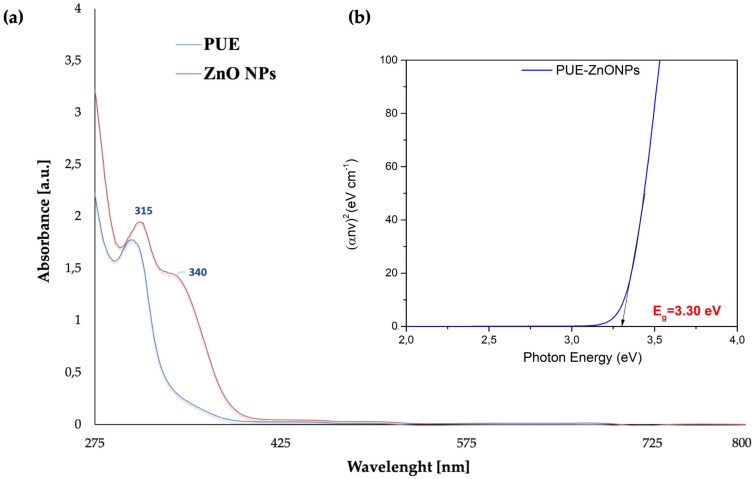
UV-vis analysis: (**a**) spectrum of puerarin (blue line) and of the green-synthesized ZnO NPs (red line); (**b**) band gap of the green-synthesized PUE-ZnO NPs.

Figure 4 displays the UV-vis spectrum of the ZnO NPs synthesized by using puerarin, recorded in the 200–800 nm range. The UV-vis spectrum revealed two absorbance peaks, at 315 and 340 nm, while the spectrum of pure puerarin showed only one peak at 302 nm, which can be assigned to the benzoyl ring of its structure. This peak was shifted to a slightly higher wavelength (bathochromic shift) when the nanoparticles were formed. The absorbance peak at 340 nm indicates that zinc oxide nanoparticles with puerarin have been formed, which is consistent with the absorbance peaks reported in previous studies and demonstrates that puerarin was involved in the reduction of Zn^2+^ [51,52,53,54].

The UV-vis spectra also provided useful information about the optical band-gap energy at various light frequencies. The empirical Tauc equation [55,56,57] was employed to expand the linear component of graph (αhν)^2^ versus optical band-gap energy (E_g_), and to calculate the band-gap energy value of the PUE-ZnO NPs, as shown in Figure 4:

(2) $${\left(\alpha h\nu \right)}^{2}=A\left(h\nu -E_{g}\right)$$

The empirical Tauc equation is characterized by its parameters, which include α—absorption coefficient, h—Planck’s constant, ν—frequency, E_g_—optical band-gap energy, and A—proportionality constant that is contingent upon the transition probability [55,57].

The PUE-ZnO NPs possess a calculated optical band-gap energy (E_g_) of 3.30 eV, which is well matched with the literature reports of ZnO NPs synthesized using different methods [58,59,60,61], but also suggests future applications as a photocatalytic nanomaterial because the photocatalytic activity is more effective when the band-gap value is lower [62,63].

#### 3.1.3. Fourier Transform Infrared (ATR-FT-IR) Spectroscopy

**Figure 5:**
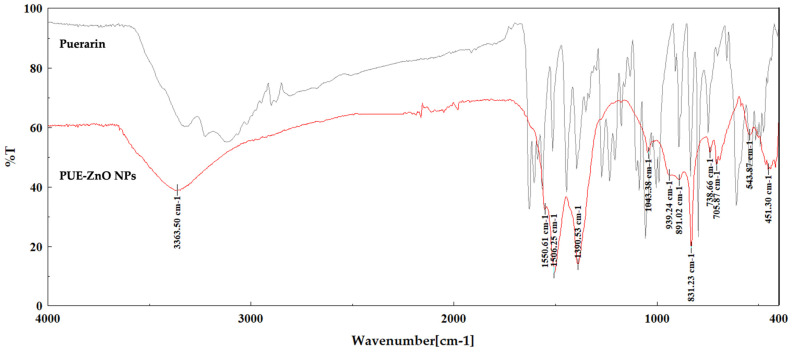
Superimposed FT-IR spectra of synthesized PUE-ZnO NPs (red) and puerarin (black).

This investigation was performed to demonstrate the formation of zinc oxide nanoparticles with the included isoflavone. Figure 5 shows the FT-IR spectra of the synthesized PUE-ZnO NPs compared to pure puerarin, in the wavenumber range of 4000–400 cm^−1^.

As also shown in Table 1, the most important peaks recorded for the analyzed nanobiocomposite were identified at 3363 cm^−1^, 1550 cm^−1^, 1506 cm^−1^, 1390 cm^−1^, 1043 cm^−1^, 831 cm^−1^, 543 cm^−1^, and 451 cm^−1^. The specific absorption bands of pure puerarin can be identified using the FT-IR spectrum shown in the Supplementary Materials (Figure S1). Despite the obvious peak broadening, the most important bands that can be assigned to the isoflavone structure are also present in the spectrum of the obtained nanoparticles, proving the presence of these functional groups in the analyzed sample.

Close results have been reported in the literature for zinc oxide nanoparticles synthesized by using other flavonoids [24,26,54,64]. The O-H stretching vibrations at 3363 cm^−1^ could be assigned to the joint contributions of the phenolic groups of the isoflavone and the hydroxy groups of the glucose moiety. At the same time, the C-H stretching vibrations bands with low intensity identified at 2899 cm^−1^ in the spectrum of puerarin are not visible in the spectrum of the composite due to the lower concentration of the isoflavone. The C=O stretching vibration at 1506 cm^−1^ in the spectrum of nanoparticles means that the aryl ketone functional group of puerarin is present in the composite. The absorption peak recorded at 1043 cm^−1^ could be ascribed to the stretching vibrations of the C-O-C functional groups in the pyran ring and in the glucoside moiety of puerarin, also demonstrating the presence of the isoflavone attached to the ZnO nanoparticles. The absorption bands at 451 cm^−1^ and 543 cm^−1^ were assigned to the symmetric bending vibration of Zn-O, based on the literature reports [65,66]. Puerarin’s reducing and stabilizing action is facilitated by the carbonyl and hydroxyl groups, resulting in the formation of zinc oxide nanoparticles, as also mentioned in previous reports concerning nanobiocomposites synthesized with different reducing agents [67,68].

#### 3.1.4. Scanning Electron Microscopy (SEM) and EDX Analysis

**Figure 6:**
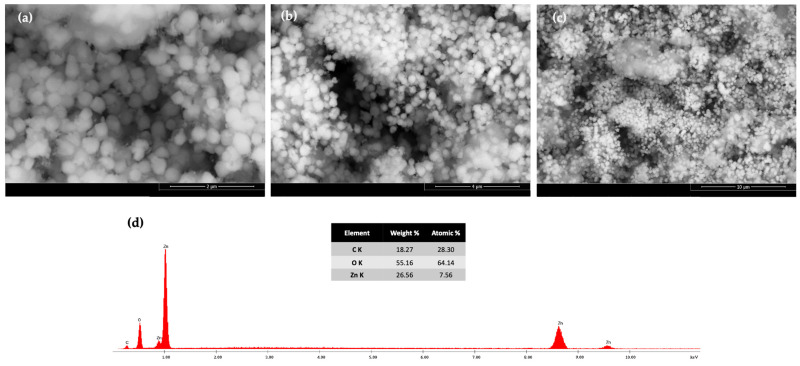
SEM analysis images of PUE-ZnO NPs obtained at different magnification: (**a**) 2 μm, 50,000×; (**b**) 4 μm, 25,000×; and (**c**) at 10 μm, 10,000×; (**d**) EDX spectra recorded for PUE-ZnO NPs.

The particle size and surface morphology of agglomerated ZnO NPs was also evaluated using SEM analysis. As shown in Figure 6, SEM analysis reveals that the nanoparticles possess a slight quasi-spherical form, with a rough surface, and the diameters of these nanoparticles are between 200 and 400 nm.

According to the EDX spectrum (Figure 6d), zinc is the dominant element with a significant and sharp peak that accounts for 26.56% of the elemental composition. Additionally, carbon and oxygen constitute 18.27% and 55.16%, respectively. Consequently, the nanoparticle`s surface contains puerarin, demonstrated by the presence of carbon and oxygen. The results also show that using our specific green synthetic approach, the resulted nanoparticles were obtained in a spherical form, which is mostly convenient for the targeted applications.

**Figure 7:**
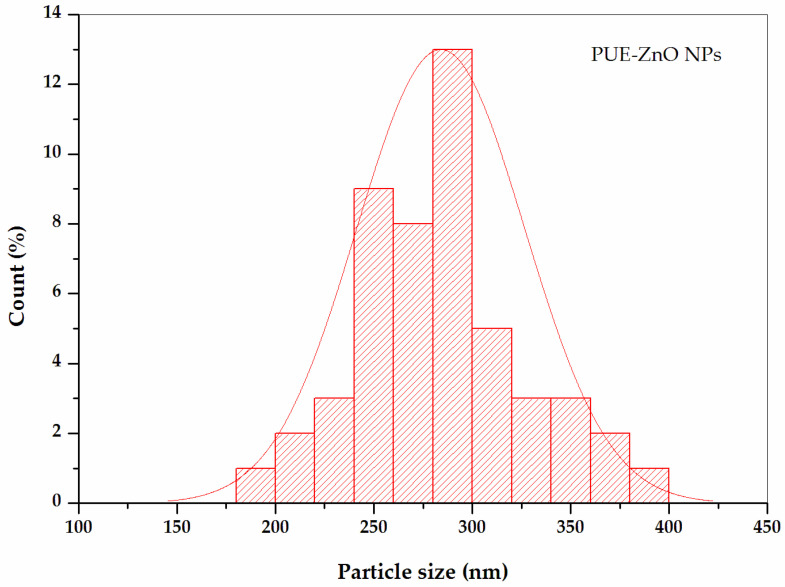
Particle size distribution histogram of PUE-ZnO NPs based on SEM analysis.

The particle size measurement was determined also by using Image-J software for at least 100 particles, based on PUE-ZnO NPs SEM images. The majority of PUE-ZnO NPs were counted in the size range between 200 and 400 nm, with a few being counted below 200 nm. The histogram for the size distribution of PUE-ZnO NPs (Figure 7), which indicates an average particle size of ~278 nm.

#### 3.1.5. Atomic Force Microscopy Analysis (AFM) of PUE-ZnO NPs

**Figure 8:**
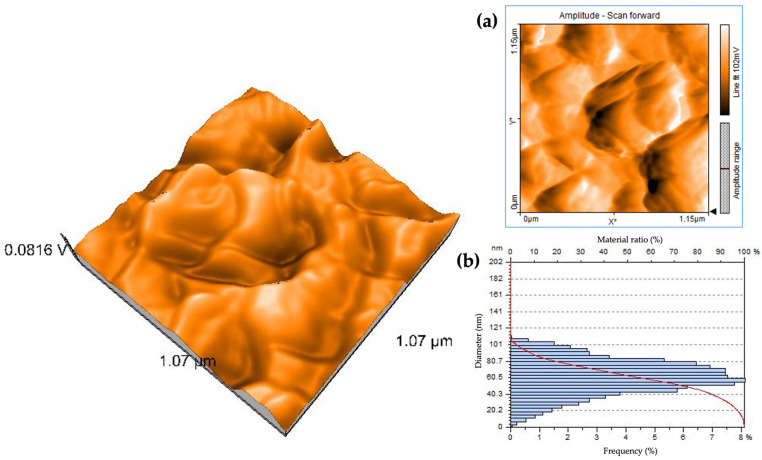
The 3D AFM image of PUE-ZnO NPs, with (**a**) 2D image and (**b**) height distribution.

Atomic force microscopy (AFM) is another technique that can provide more detailed information on the morphological characteristics of nanoparticles, and it has become a powerful tool for nanotechnology [68,69]. Structural information on the PUE-ZnO NPs can be obtained through AFM and SEM analyses data. The AFM images for PUE-ZnO NPs are shown in Figure 8.

The size of the agglomerates varies between 40 and 80 nanometers. The surface roughness (Sa) of the PUE-ZnO NPs is 28,941 nm. The valley depth (Sv) has a minimum value of −100.5 nm, and the maximum peak height (Sp) is 85,294 nm. The surface area is large and rough due to the attachment of puerarin, which is aided by these triangle-type formations. The arithmetic average roughness (Ra) has a value of 37 nm, and the line roughness parameter (Rq) value is 42 nm. These results are consistent with the SEM analysis data (Figure 6).

### 3.2. Antimicrobial Potential of Green-Synthesized PUE-ZnO NPs

ZnO nanoparticles have become a significant topic over time due to their diverse medical applications, which include antimicrobial properties. The antimicrobial potential of PUE-ZnO NPs was evaluated determining the minimum inhibitory concentration (MIC), using the broth dilution method on three different bacterial strains: (i) two Gram-negative bacteria (*Escherichia coli*, *Pseudomonas aeruginosa*); (ii) two Gram-positive bacteria (*Staphylococcus aureus*, *Streptococcus pyogenes*); and (iii) one yeast strain (*Candida parapsilosis*).

According to Table 2, PUE-ZnO NPs showed the lowest antibacterial activity (MIC = 50 mg/mL) against *Candida parapsilosis*, while the highest antibacterial activity (MIC = 25 mg/mL) was recorded at the same value against the two Gram-positive and one Gram-negative (*Escherichia coli*) bacterial strains. The MIC value could not be determined for the *Pseudomonas aeruginosa* strain, which may be due to its natural resistance to inactivation by most traditional antibiotics [54,70,71].

### 3.3. In Ovo Assay

#### 3.3.1. The Anti-Irritant Effect by PUE-ZnO NPs Using the HET-CAM Method

**Figure 9:**
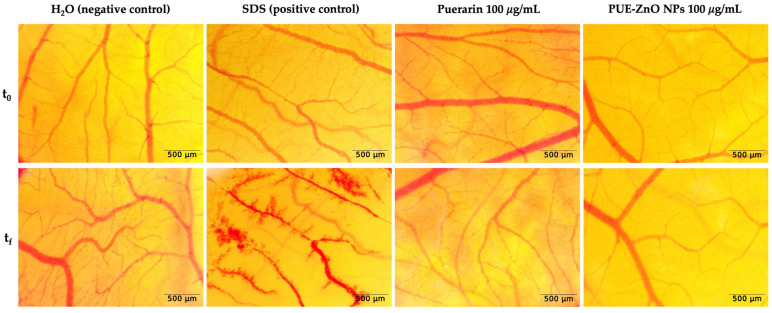
Representative images of PUE-ZnO nanoparticles evaluated using the HET-CAM method. Stereomicroscopic images of the chorioallantoic membrane after treatment with H_2_O (negative control), SDS 0.5% (positive control), and test samples at a concentration of 100 μg/mL; images represent the CAM area of administration before sample application (t_0_) and five minutes after application (t_f_), by stereomicroscopy, 3.2× magnification.

The evaluation of the in ovo potential irritative effect was performed using the HET-CAM method, by calculating and evaluation by the irritation score (IS) for every sample and control group. According to Table 3, the positive control had a significant irritant effect (IS = 17.29), while the negative control, puerarin and PUE-ZnO nanoparticles, did not cause any irritation (IS = 0); thus, they do not present any irritation effect. Figure 9 illustrates that when the samples and controls were applied to the chorioallantoic membrane, the positive control revealed all the vascular events, while the negative control, puerarin and PUE-ZnO nanoparticles, did not experience any vascular events.

Our HET-CAM method results showed that PUE-ZnO nanoparticles (IS = 0 and classified as non-irritant by Luepke’s criteria), when evaluated at a concentration of 100 µg/mL, revealed a possible safety level on the chorioallantoic membrane. To achieve a comprehensive in ovo safety profile, the long-term effects of repeated exposure of the PUE-ZnO NPs on the membrane is needed. Further in our study, the effect of these nanoparticles on angiogenesis was studied.

#### 3.3.2. Modulation of Angiogenesis by PUE-ZnO NPs Using CAM Assay

The investigation of potential effects of ZnO nanoparticles on angiogenesis [72,73] was accomplished through the use of the chorioallantoic membrane (CAM) assay, which is a highly effective method that can be performed easily and cost effectively, allowing it to be used as a complementary test for studying the biological effects of nanoparticles and nanomaterials [74,75].

**Figure 10:**
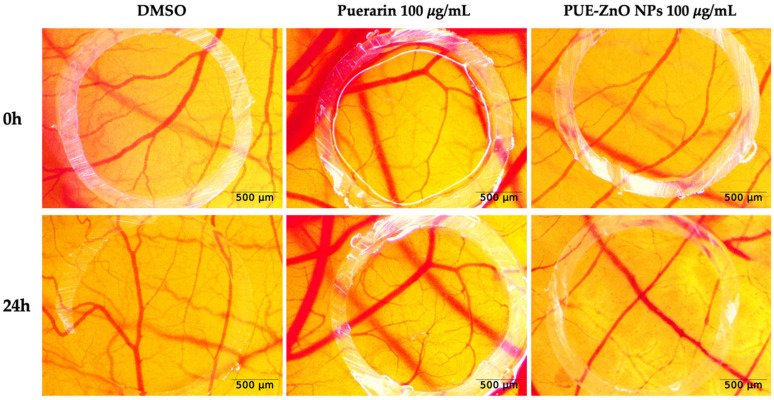
The angiogenesis assessment of PUE-ZnO nanoparticles, using the CAM assay. Stereomicroscope images represent the 24 h modification upon the treated vascular plexus; scale bars represent 500 µm.

In a similar manner to the previous method, we applied DMSO, puerarin, and PUE-ZnO NPs to the chorioallantoic membrane and monitored stereomicroscopically to identify if any inhibition of the angiogenesis process was possible. After 24 h post-treatment, no relevant modifications or anomalies were observed concerning the normal developing vessel architecture, which indicates that the angiogenesis process was not affected (Figure 10). Thus, it can be concluded that ZnO nanoparticles synthesized through a green chemistry pathway, at a concentration of 100 μg/mL, exhibit good vascular tolerance.

## 4. Conclusions

In this study, the isoflavone puerarin was used for the first time to synthesize ZnO NPs by a green chemistry approach. This new hybrid material was obtained through a very friendly synthetic route without any other chemicals and at a much lower temperature compared to similar approaches. The formation of the nanoparticles was highlighted by the UV-vis spectra, with the apparition of a maximum absorption band at 340 nm. The band gap for the PUE-ZnO nanoparticles is very sharp, with a calculated value of 3.30 eV, suggesting a high potential for photocatalytic activity. Structural analysis by FT-IR also revealed the effective synthesis of ZnO composite nanoparticles mediated by puerarin. The high purity and crystalline nature of PUE-ZnO NPs was confirmed by the XRD study, which also proves the success of the green synthetic pathway. The quasi-spherical morphology and size of the green-synthesized PUE-ZnO NPs between 200 and 400 nm, were confirmed by SEM analysis, while the detailed surface morphology was assessed by AFM. Due to their antimicrobial potential, as well as their non-irritating and angiogenesis-limiting properties, the ZnO nanoparticles synthesized by using puerarin as a reducing and stabilization agent are a promising option for the hybrid nanoparticle field. The present work was designated to demonstrate the possibility of developing a greener pathway for the synthesis of metal oxide–flavonoid nanoparticle composites, and its success will allow the forthcoming investigation of their therapeutic and personal care applications.

## Figures and Tables

**Table 1:** FT-IR spectra with potential assignments for both PUE-ZnO NPs and puerarin.

Possible Assignment for Compound Class	Absorption Peaks in PUE-ZnO NPs (cm^−1^)	Absorption Peaks in Puerarin (cm^−1^)
O-H stretching vibrations	3363	3324
	-	3224
	-	3118
	-	2899
C-C bond stretching of the aromatic rings	1550	1564
C=O stretching vibrations of the aryl ketone group	1506	1514
O-H bending in-plane vibrations of the phenolic groups	1390	1396
C-O-C stretching vibrations	1043	1057
C-H aromatic bending vibrations with substituted arrays	831	835
Zn-O symmetric vibrations	543	-
	451	-

**Table 2:** The minimum inhibitory concentration of PUE-ZnO NPs for the tested strains.

Bacterial and Yeast Strains	MIC Value (mg/mL)
*Staphylococcus aureus* ATCC 25923	25
*Streptococcus pyogenes* ATCC 19615	25
*Escherichia coli* ATCC 25922	25
*Pseudomonas aeruginosa* ATCC 27853	NA
*Candida parapsilosis* ATCC 22019	50

NA—no activity.

**Table 3:** Irritability evaluation using the HET-CAM assay for puerarin and PUE-ZnO NPs (in concentration of 100 µg/mL) and control samples (distilled water, SLS 0.5%).

Test Compound and Controls	Irritation Score (IS)	* Irritation Category/Type of Effect
**Negative control H_2_O**	0	Non-irritant
**Positive control SDS 0.5%**	17.29	Strongly irritant
**Puerarin, 100 μg/mL**	0	Non-irritant
**PUE-ZnO NPs, 100 μg/mL**	0	Non-irritant

* Luepke scale: non-irritant (0 ÷ 0.9), weak irritant (1 ÷ 4.9), moderately irritant (5 ÷ 8.9), strongly irritant (8.9 ÷ 21) [72].

## Data Availability

Data are contained within the article.

## Supplementary Materials

The following Supporting Information can be downloaded at: https://www.mdpi.com/article/10.3390/pharmaceutics16111464/s1, Figure S1: FT-IR spectrum of Puerarin.
