# Supplementary material for: Green Synthesis of Zinc Oxide Nanoparticles Using Puerarin: Characterization, Antimicrobial Potential, Angiogenesis, and In Ovo Safety Profile Assessment
Source: Pharmaceutics. 2024 Nov 16;16(11):1464. doi: 10.3390/pharmaceutics16111464 (PMC11597859; doi:10.3390/pharmaceutics16111464)
Supplement: Supplementary file 1 [file pharmaceutics-16-01464-s001.zip › pharmaceutics-3276285-supplementary.pdf]

Article

Green Synthesis of Zinc Oxide Nanoparticles Using Puerarin: Characterization, Antimicrobial Potential, Angiogenesis, and In Ovo Safety Profile Assessment

Sergio Liga [1], Raluca Vodă [2], Lavinia Lupa [2], Cristina Paul [1,*], Nicoleta Sorina Nemeş [3], Delia Muntean [4], Ștefana Avram [5], Mihaela Gherban [6] and Francisc Péter [1,3]

1 Department of Applied Chemistry and Engineering of Organic and Natural Compounds, Faculty of Chemical Engineering, Biotechnologies and Environmental Protection, Politehnica University Timisoara, Vasile Pârvan No. 6, 300223 Timisoara, Romania; sergio.liga96@gmail.com (S.L.); francisc.peter@upt.ro (F.P.)

2 Department of Applied Chemistry and Environmental Engineering and Inorganic Compounds, Faculty of Chemical Engineering, Biotechnologies and Environmental Protection, Politehnica University Timisoara, Vasile Pârvan No. 6, 300223 Timisoara, Romania; raluca.voda@upt.ro (R.V.); lavinia.lupa@upt.ro (L.L.)

3 Renewable Energy Research Institute-ICER, Politehnica University Timisoara, Gavril Musicescu Street No. 138, 300501 Timisoara, Romania; nicoleta.nemes@upt.ro

4 Multidisciplinary Research Center on Antimicrobial Resistance, Department of Microbiology, Faculty of Medicine, "Victor Babes, " University of Medicine and Pharmacy, 2nd Eftimie Murgu Sq., 300041 Timisoara, Romania; muntean.delia@umft.ro

5 Department of Pharmacognosy, Faculty of Pharmacy, "Victor Babes, " University of Medicine and Pharmacy, Eftimie Murgu Square, No. 2, 300041 Timisoara, Romania; stefana.avram@umft.ro

6 National Institute for Research and Development in Electrochemistry and Condensed Matter, P. Andronescu Street, No. 1, 300224 Timisoara, Romania; mihaelabirdeanu@gmail.com

* Correspondence: cristina.paul@upt.ro

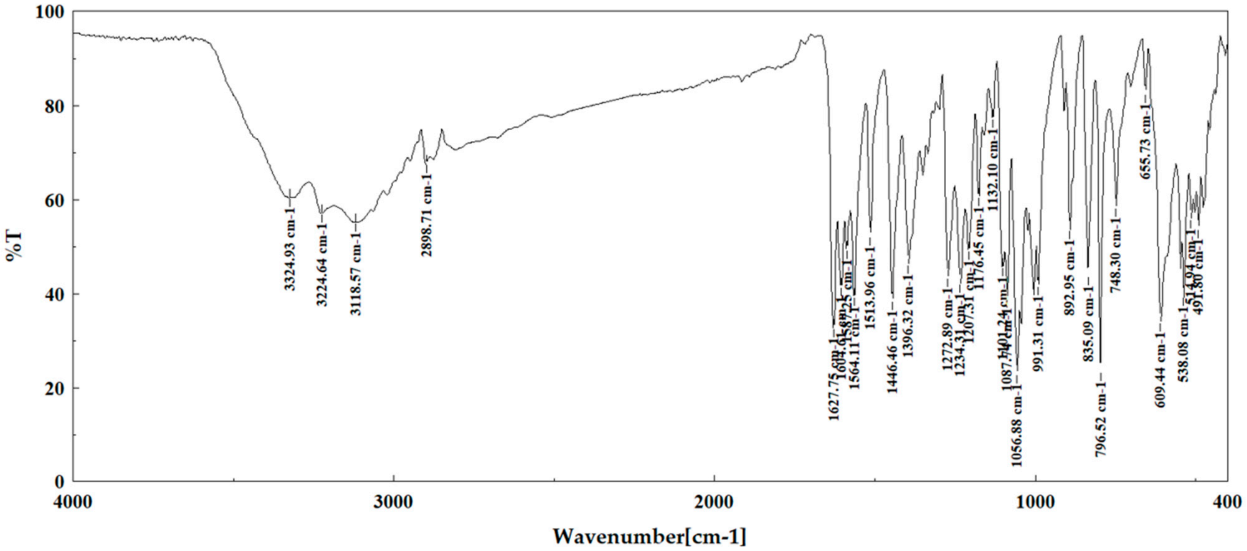

Figure S1. FT-IR spectrum of Puerarin
